# The clinical practice and outcomes of minimally invasive surgery in primary malignant melanoma of the vagina and cervix patients: a retrospective cohort study

**DOI:** 10.1186/s13023-025-03760-x

**Published:** 2025-06-06

**Authors:** Bin Liu, Yan Liu, Haixin He, Wei Chen, Haizhou Ji, Ling Lin, Quping Tan, Yang Sun, Cuibo Lin

**Affiliations:** 1 Department of Gynecology, Clinical Oncology School of Fujian Medical University, Fujian Cancer Hospital, 420 Fuma Road, Fuzhou, 350014 Fujian China; 2 Fujian Provincial Key Laboratory of Brain Aging and Neurodegenerative Diseases, School of Basic Medical Sciences, Fujian Medical University, Xue Yuan Road, University Town, FuZhou, 350108 Fujian China

**Keywords:** Rare diseases, Primary malignant melanoma of the vagina, Primary malignant melanoma of the cervix, Minimally invasive surgery, Outcomes

## Abstract

**Background:**

Primary malignant melanoma of the vagina (PMMV) and cervix (PMMC) are extremely rare tumors with a poor prognosis, lacking well-defined protocols or standardized treatment guidelines. While the preferred management for early-stage PMMV and PMMC is surgery, comprehensive reports on the impact of various surgical techniques on cancer outcomes are scarce.

**Objective:**

This study aimed to compare outcomes between open and minimally invasive surgery (MIS) in PMMV and PMMC, and concurrently evaluated prognostic risk factors for these conditions.

**Methods:**

We conducted a single-center retrospective cohort study of PMMV and PMMC patients treated surgically from January 2000 to July 2021. Clinicopathological features and surgical outcomes were assessed retrospectively. Patients underwent either open surgery or MIS. Disease-Free Survival (DFS) rates were compared.

**Results:**

Of 45 eligible patients, the MIS group showed a higher rate of total vaginectomy (*P* = 0.022), reduced median intraoperative blood loss (*P* = 0.031), shorter median hospital stay (*P* = 0.042), and no significant increase in perioperative complications (*P* = 0.867). The incidence of negative margins < 1 cm was significantly lower in the MIS group (*P* = 0.032). Cox proportional hazards regression identified microsatellites (HR = 2.893 [1.042–8.029]; *P* = 0.042), surgical negative margin distance (HR = 0.042 [0.008–0.217]; *P* < 0.001), and total vaginectomy (HR = 0.042 [0.008–0.217]; *P* < 0.001) as independent prognostic factors for DFS. MIS was linked to a significant difference in 2-year DFS (*P* = 0.030), but there was no notable difference in overall survival (OS) compared to open surgery (*P* = 0.078). The outcomes are validated through sensitivity analysis and hierarchical assessment, leading to the development of a novel nomogram simultaneously.

**Conclusions:**

Total vaginectomy may improve DFS in PMMV and PMMC patients. A combination of MIS and radical vaginal resection can effectively manage PMMV and PMMC as an initial surgical strategy.

**Supplementary Information:**

The online version contains supplementary material available at 10.1186/s13023-025-03760-x.

## Introduction

Female genital tract malignant melanoma is extremely rare, representing only 3% of all malignant melanomas [1]. This malignancy is categorized into vulvar, vaginal, and cervical melanoma based on the primary tumor location. The 5-year survival rate for cutaneous melanoma is approximately 98%, yet the prognosis for female genital tract melanoma is significantly worse [2]. Vulvar malignant melanoma shows a 5-year survival rate of 24–77%, while vaginal and cervical melanomas have even lower rates, between 5 and 25% [2–5]. The high recurrence rate of PMMV and PMMC presents a significantly greater challenge for clinicians in comparison to vulvar malignant melanoma.

Currently, no standardized treatment guidelines exist for PMMV and PMMC. Treatment largely draws from experiences with cutaneous malignant melanomas. However, most reports on treatments are case studies with variable outcomes. Surgical intervention, often paired with chemoradiotherapy and targeted therapy, remains the primary treatment approach [6–8]. Yet, detailed reports on the effects of different surgical methods on cancer outcomes are scarce.

Minimally invasive surgery (MIS), known for smaller incisions, reduced intraoperative bleeding, faster postoperative recovery, and shorter hospital stays, is increasingly used in early-stage gynecological cancers [9]. Studies have shown that MIS does not adversely affect recurrence rates and overall survival in certain cancers, like early-stage endometrial and lung cancer [10, 11]. Nonetheless, research on MIS for PMMV and PMMC is limited.

This study aimed to compare the outcomes of open surgery and MIS in PMMV and PMMC, while also examining prognostic risk factors for these conditions. The findings may guide the development of initial surgical protocols for PMMV and PMMC.

## Materials and methods

This retrospective cohort study was conducted in a major oncology hospital, focusing on patients with malignant melanoma in the female genital tract who underwent initial surgery from January 2000 to July 2021. The study received approval from the hospital’s ethics committee (K2023-246-01) and adhered to the Declaration of Helsinki’s principles.

### Patient selection

**Fig. 1:**
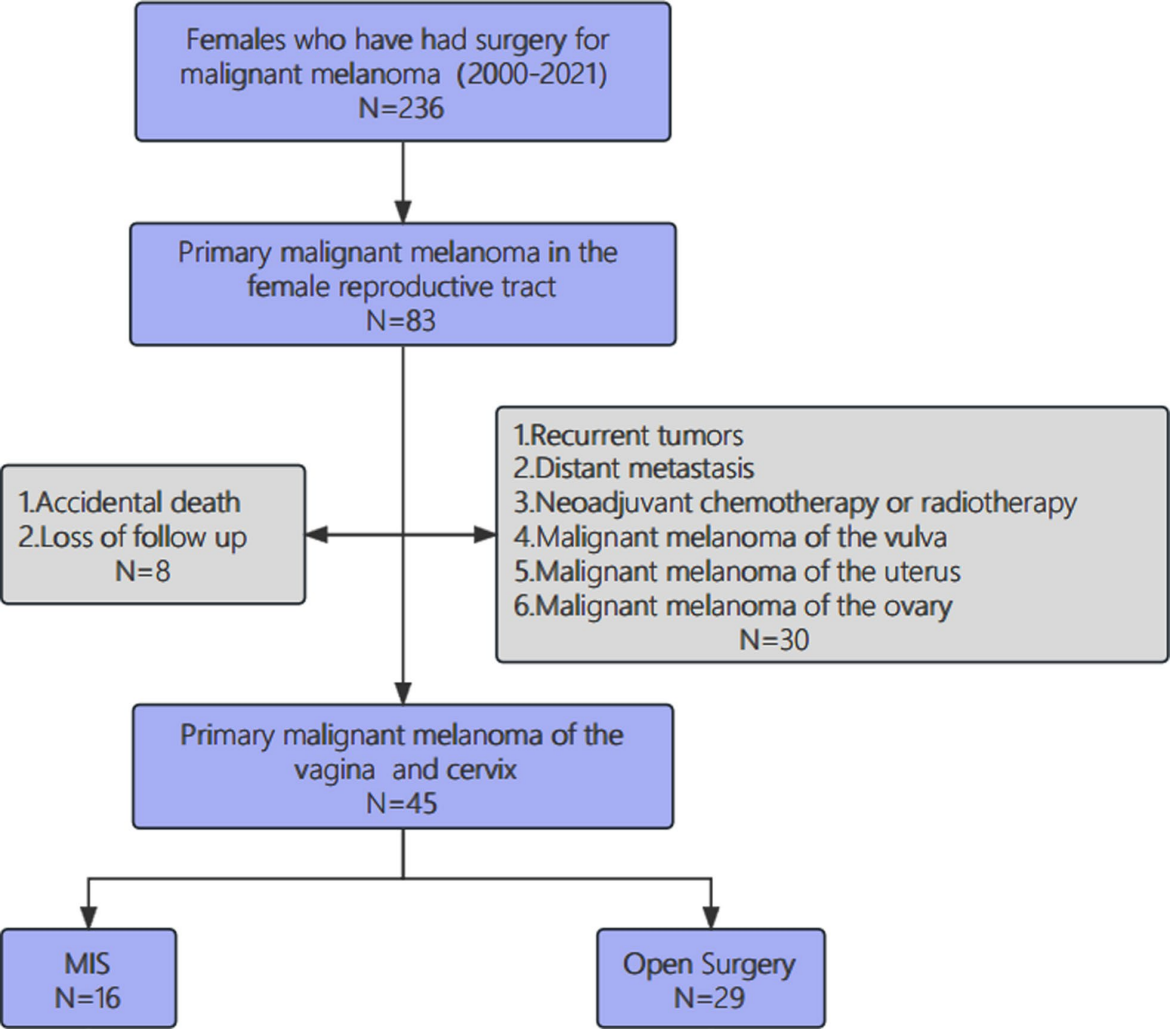
Flow chart of patients selection

All patients provided written consent for their electronic medical records and biospecimens to be accessed (the process of informed consent was completed upon admission). Clinicopathological data were obtained from the electronic medical record system, including age, weight, comorbidities, tumor stage, histologic type, surgical procedure, surgery extent, tumor location and size, lymph node metastasis status, mitotic count, and adjuvant therapy. The study excluded cases of primary malignant melanoma of the uterus, ovaries, and vulva. In instances where the melanoma involved both the vulva and vagina, the primary tumor was identified based on the site with the largest tumor diameter. Cases with distant metastasis, recurrence, or prior neoadjuvant chemoradiotherapy were also excluded. Additionally, 8 patients lost to follow-up or with unconfirmed survival status (inconsistencies in the survival status reported during the telephone follow-up) were removed from the study. The patient selection process is detailed in the flow chart (Fig. 1).

### Procedures

The preoperative evaluation for each patient included MRI or PET-CT scans to exclude distant metastases. The choice between open surgery and minimally invasive surgery (MIS) was made jointly by the attending physician and the patient. Surgeons with over ten years of experience in gynecological malignant tumor surgery performed the operations.

Surgical procedures included radical or simple hysterectomy ± systematic lymph node dissection. Inguinal lymph node dissection may be performed in cases of vaginal melanoma with vulvar extension. Total vaginectomy could involve additional procedures such as vulvectomy and partial urethrectomy. For complex surgical cases, a multidisciplinary team (MDT) comprising gynecologic oncologists, medical oncologists, surgical oncologists, and anesthesiologists developed treatment strategies together. The initial postoperative follow-up was scheduled at 1 month, followed by evaluations every 2–3 months, tailored to the disease’s unique characteristics. Follow-up included symptom assessment, gynecological examination, and imaging. Recurrence determination was based on imaging or tissue biopsy pathology. Follow-up for surviving patients was at least 2 years, excluding those who died from tumor recurrence within this period.

### Data analysis and definitions

Disease-free survival (DFS) was defined as the time from surgery to the first sign of disease progression. Overall survival (OS) was the time from surgery to disease-related death, death for other causes or the last follow-up.

Clinical data were analyzed using descriptive statistics. Continuous variables between open and MIS groups were compared using Student’s t-test or the Mann–Whitney U test. Categorical variables were analyzed using Pearson’s chi-square test and Fisher’s exact test. The Cox proportional hazards regression model was used for univariate and multivariate analyses to assess impact on recurrence. Univariate analysis results with a *p*-value < 0.05 were included in multivariate analysis. The model’s resilience was additionally confirmed by accounting for potential confounding variables, subsequent hierarchical examination, and conducting sensitivity analysis. The initial model is adjusted based on the lesion’s location. Given that tumor invasion in the lower 1/3 of the vagina may result in a negative incisal margin of less than 1 cm, our study population for the first model was limited to individuals with tumor invasion in the upper 2/3 of the vagina. Additionally, it should be noted that surgical outcomes can be influenced by the location of the lesion. In Model 2, patients who did not receive standardized adjuvant therapy following surgery were excluded. This includes individuals with inadequate treatment duration or those who did not undergo radiotherapy or chemotherapy. Failure to adhere to the National Comprehensive Cancer Network (NCCN) guidelines for standard postoperative adjuvant therapy may lead to a higher risk of short-term tumor recurrence. Finally, in order to validate the significant factors with a limited sample size, we utilized the bootstrap method by conducting 1000 samplings. This approach involved multiple iterations of sampling, evaluating model performance for each iteration, and consolidating outcomes to corroborate or enhance initial conclusions regarding single-factor and multi-factor analysis based on verification results.

The Kaplan–Meier method and log-rank test compared the effects of different surgical techniques and total vaginectomy on survival outcomes. A novel nomogram was created by integrating the results of multiple models alongside clinical expertise, and its predictive accuracy was assessed using the Bootstrap-Receiver Operating Characteristic (BS-ROC) curve and BS calibration curve based on the BS validation method. Statistical significance was determined with a P-value below 0.05. Data analysis and visualization were conducted utilizing R package (version 4.1.0) and GraphPad Prism (version 8.0).

## Results

In this study, 45 patients meeting the inclusion criteria were identified, with 16 (35.6%) undergoing MIS and 29 (64.4%) opting for open surgery. The demographic and clinicopathological data of all patients are presented in Table 1. The median age was similar in both groups, with 53 years in the MIS group and 54 years in the open group. There was a significant difference in median BMI values between the MIS group and the open group (23.8 kg/m2 vs. 21.6 kg/m2; P < 0.05). No significant differences were observed between the groups in terms of comorbidities, histological subtypes, AJCC stage, surgery extent, lymph node metastasis, lesion location, tumor number, mitotic count, microsatellites, or adjuvant therapy. The most common histological subtype was nodular (50.0%), followed by Superficial spreading (18.8%). The superficial spreading subtype typically appeared as speckled black lesions, mainly affecting the lower third of the vagina and labia minora, while the nodular subtype predominantly involved the upper third of the vagina and cervix. Multiple lesions were more prevalent than single lesions, with microsatellites (48.9%) detected in nearly half of the cases.

**Table 1:** Clinopathological characteristics of patients

Variable	MIS (n = 16)	Open (n = 29)	*P*-value
Age, year,median (range)	53(42,86)	54(39,75)	0.768
BMI, kg/m^2^,median (range)	23.8(19.9,31.2)	21.6(18.7,30.3)	0.010*
Comorbidity,%			0.271
Yes	7(43.6%)	8(27.6%)	
No	9(56.3%)	21(72.4%)	
Histologic subtype,%			0.989
Nodular	8(50.0%)	13(44.8%)	
Superficial spreading	3(18.8%)	8(27.6%)	
Amelanotic	1(6.3%)	2(6.9%)	
Spindle cell	2(12.5%)	5(17.2%)	
Other	2(12.5%)	1(3.4%)	
Lymphadenectomy,%			0.292
Yes	14(87.5%)	21(72.4%)	
No	2(12.5%)	8(27.6%)	
AJCC stage,%			0.528
I	5(31.3%)	12(41.4%)	
II	9(56.3%)	5(17.2%)	
III	2(12.5%)	12(41.4%)	
Surgery,%			0.630
RH	6(37.5%)	13(44.8%)	
SH	10(62.5%)	16(55.2%)	
LN metastasis,%			0.096
Yes	2(12.5%)	12(41.4%)	
No	14(87.5%)	17(58.6%)	
Mitotic count,%			0.360
< 10	6(37.5%)	15(51.7%)	
≥ 10	10(62.5%)	14(48.3%)	
Lesion localization(Vagina),%			0.474
Upper 1/3	7(43.8%)	14(48.3%)	
Middle 1/3	3(18.8%)	9(31.0%)	
lower 1/3	6(37.5%)	6(20.7%)	
Number of tumors (macroscopic),%			0.339
Single	7(43.8%)	17(58.6%)	
Multiple	9(56.3%)	12(41.4%)	
Microsatellites,%			0.608
Yes	7(43.8%)	15(51.8%)	
No	9(56.3%)	14(48.3%)	
Adjuvant therapy,%			0.322
Yes	13(81.3%)	19(65.5%)	
No	3(18.8%)	10(34.5%)	

*Statistically signifcant MIS: minimally invasive surgery, BMI: body mass index, RH: radical hysterectomy, SH: simple hysterectomy

The median follow-up duration was longer in the MIS group compared to the open surgery group (24.5 months vs. 12 months), but this difference was not statistically significant. The MIS group had comparable operative times, intraoperative blood loss, and perioperative complications to the open group. Furthermore, the MIS group exhibited a significantly higher incidence of total vaginectomy (62.5%) compared to the open group (27.6%) (*P* = 0.022) and also achieved longer negative surgical margins (*P* = 0.032). The 2-year recurrence rate was 65.5% in the MIS group, while it was 31.3% in the open group (*P* = 0.027) (Table 2). Patients who underwent total vaginectomy were further classified into two subgroups: the MIS cohort and the open cohort. Significant differences were observed between these cohorts in terms of intraoperative blood loss and hospitalization duration, with the MIS cohort showing significantly lower values compared to the open cohort (*P* = 0.031; *P* = 0.042). Both cohorts experienced grade 2–3 perioperative complications (*P* = 0.619). There was no significant difference in 30-day readmission rates between the MIS cohort (n = 1; 10.0%) and the open cohort (n = 2; 25.0%). The 2-year recurrence rate for patients undergoing total vaginectomy did not show a statistically significant difference (*P* = 0.832) (Table 3).

**Table 2:** Surgical outcomes of patients

Variable	MIS (n = 16)	Open (n = 29)	*P*-value
Duration of surgery, minute, median (range)	230(60,540)	195(80,500)	0.240
Intraoperative blood loss,ml, median (range)	200(100,800)	300(100,1000)	0.381
Surgical Margin			0.032^*^
≤ 1 cm	1(6.25%)	12(41.4%)	
> 1 cm	15(93.75%)	17(58.6%)	
*Total vaginectomy,%*			
Yes	10(62.5%)	8(27.6%)	0.022^*^
No	6(37.5%)	21(72.4%)	
Recurrences(2 years), %			0.027*
Yes	5(31.25%)	19(65.5%)	
No	11(68.75%)	10(34.5%)	
*Postoperative Complications,%*			
Yes	4(25.0%)	6(20.7%)	0.967
No	12(75.0%)	23(79.3%)	
Follow-up time, months, median (range)	24.5(3.3–40.0)	12(5.3–30.0)	0.104

*Statistically signifcant MIS minimally invasive surgery

**Table 3:** Surgical and perioperative parameters and survival outcomes of patients with total vaginectomy

Variable MIS Open *P*-value (n = 10) (n = 8)			
Duration of surgery, minute,median (range)	300(200,540)	550(190,420)	0.531
Intraoperative blood loss, ml,median (range)	300(250,800)	550(200,1000)	0.031*
Length of stay days,median (range)	9(6,14)	12(8,20)	0.042^*^
Postoperative Complications,%			0.867
Yes	3(30.0%)	3(37.5%)	
No	7(70.0%)	5(62.5%)	
Highest complication grade,%			0.619
1	0	0	
2	2(20.0%)	1(12.5%)	
3	1(10.0%)	2(25.0%)	
Readmission within 30 days,%	1(10.0%)	2(25.0%)	0.832
Recurrences(2 years),%	1(10.0%)	2(25.0%)	0.832
Follow-up time, months,median (range)	29.0(25.6–40.0)	24.5(6.2–36.7)	0.099

*Statistically signifcant

The Cox proportional hazards regression model was used to assess risk factors associated with DFS. Univariate analysis showed that the type of hysterectomy (HR = 0.410 [0.169–0.992]; *P* = 0.041), surgical approach (HR = 2.850 [1.061–7.652]; *P* = 0.038), total vaginectomy (HR = 9.464 [2.784–32.176]; *P* < 0.001), length of surgical margin (HR = 0.023 [0.005–0.107]; *P* < 0.001), and microsatellite metastasis (HR = 3.120 [1.321–7.366]; *P* = 0.001) were significant risk factors for DFS. In multivariate analysis, only total vaginectomy (HR = 0.155 [0.037–0.641]; *P* = 0.010), length of surgical margin (HR = 0.042 [0.008–0.217]; *P* < 0.001), and microsatellite metastasis (HR = 2.893 [1.042–8.029]; *P* = 0.042) remained significant (Table 4). In stratification and sensitivity analysis, Model 1 was adjusted to control for lesion location, specifically excluding patients with lesions located in the lower third of the vagina. The findings revealed that total vaginectomy, microsatellites, and adjuvant therapy were identified as significant risk factors for DFS (*P* < 0.05) (Table S1). We subsequently conducted a reanalysis of DFS in a subset of patients who underwent standard adjuvant therapy following surgery, reaffirming our primary findings that total vaginectomy, microsatellites, and surgical margin length remain significant risk factors influencing DFS (*P* < 0.05) (Table S2). The result obtained was subjected to bootstrap statistical analysis and subsequently verified using 1000 random samples. This validation process yielded a C-index value of 0.973, accompanied by a 95% confidence interval spanning from 0.718 to 0.916.

**Table 4:** Multivariate cox regression analysis of DFS

Characteris	DFS						
	Univariate	Analysis			Multivariate	Analysis	
	HR	95%CI	*P*-value		HR	95%CI	*P*-value
*Age, year*							
(< 55 vs. ≥ 55)	0.820	0.364–1.847	0.632				
*BMI, kg/m2*							
(< 22 vs. ≥ 22)	0.987	0.438–2.223	0.974				
*Comorbidity*							
(Yes vs. No)	0.778	0.340–1.780	0.552				
*Type of hysterectomy*							
(SH vs. RH)	0.410	0.169–0.992	0.041^*^	0.508	0.183–1.412	0.194	
Lymphadenectomy							
(Yes vs. No)	2.144	0.916–5.021	0.079				
*LN metastasis*							
(Yes vs. No)	1.715	0.749–3.926	0.201				
AJCC stage (I or II vs.III)	0.832/1.586	0.296–2.337 0.629–4.003	0.726 0.329				
*Mitotic count*							
(< 10 vs. ≥ 10)	0.781	0.349–1.745	0.781				
*Surgical approach*							
(MIS vs. Open)	2.850	1.061–7.652	0.038^*^	1.833		0.638–5.267	0.261
*Total vaginectomy*							
(Yes vs. No)	9.464	2.784–32.176	0.000^*^	0.155		0.037–0.641	0.010^*^
*Histologic subtype*							
(Other vs.Spreading And Nodular)	1.028/1.302	0.380–2.780 0.437–3.880	0.957 0.635				
*Surgical Margin*							
(≤ 1 cm vs. > 1 cm)	0.023	0.005–0.107	0.000*	0.042	0.008–0.217	0.000^*^	
*Lesion localization*							
(Upper vs. Middle And Lower)	0.670/0.324	0.257–1.747 0.107–1.000	0.412 0.050				
*Number of tumors*							
(Single vs. Multiple)	0.766	0.342–1.714	0.517				
Microsatellites							
(Yes vs. No)	3.120	1.321–7.366	0.001^*^	2.893		1.042–8.029	0.042^*^
*Adjuvant therapy*							
(Yes vs. No)	1.431	0.633–3.237	0.390				

*Statistically signifcant DFS disease-free survival, MIS minimally invasive surgery, BMI body mass index, RH radical hysterectomy, SH simple hysterectomy

**Fig. 2:**
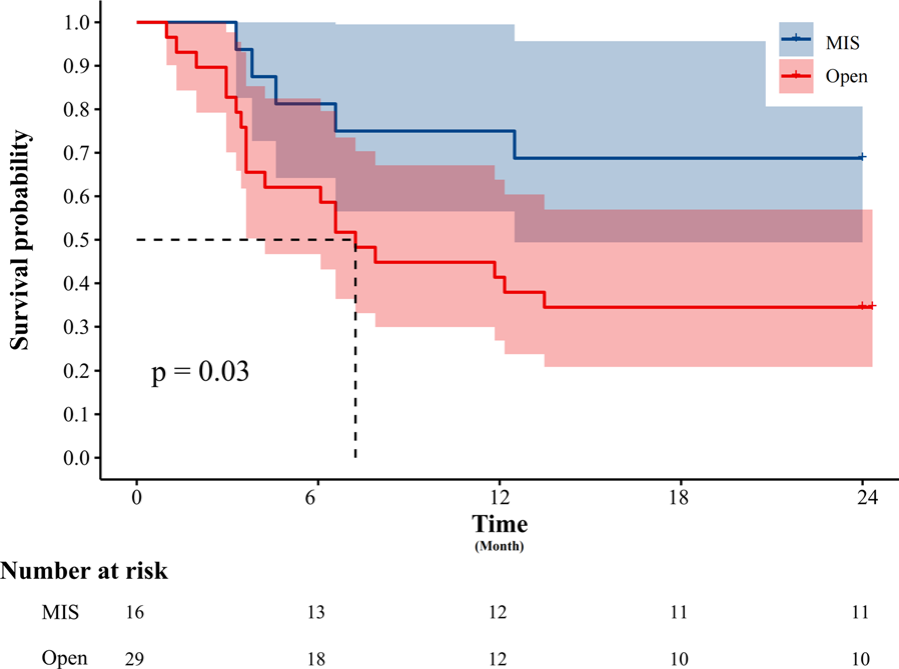
The Kaplan–Meier survival curve demonstrates the 2-year disease-free survival rates of patients with primary malignant melanoma of the vagina and cervix who underwent minimally invasive surgery

**Fig. 3:**
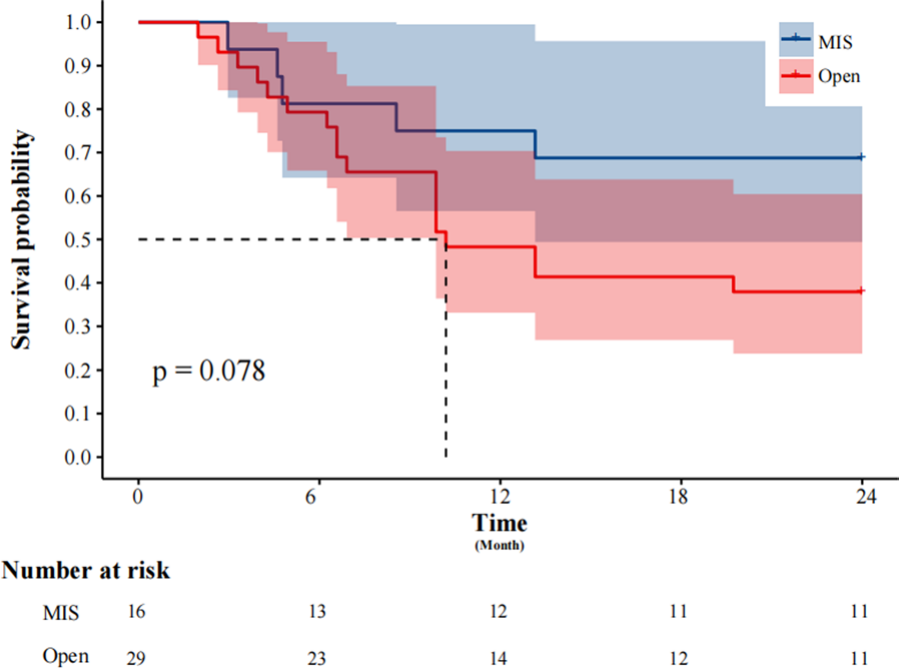
The Kaplan–Meier survival curve demonstrates the 2-year overall survival rates of patients with primary malignant melanoma of the vagina and cervix who underwent minimally invasive surgery

**Fig. 4:**
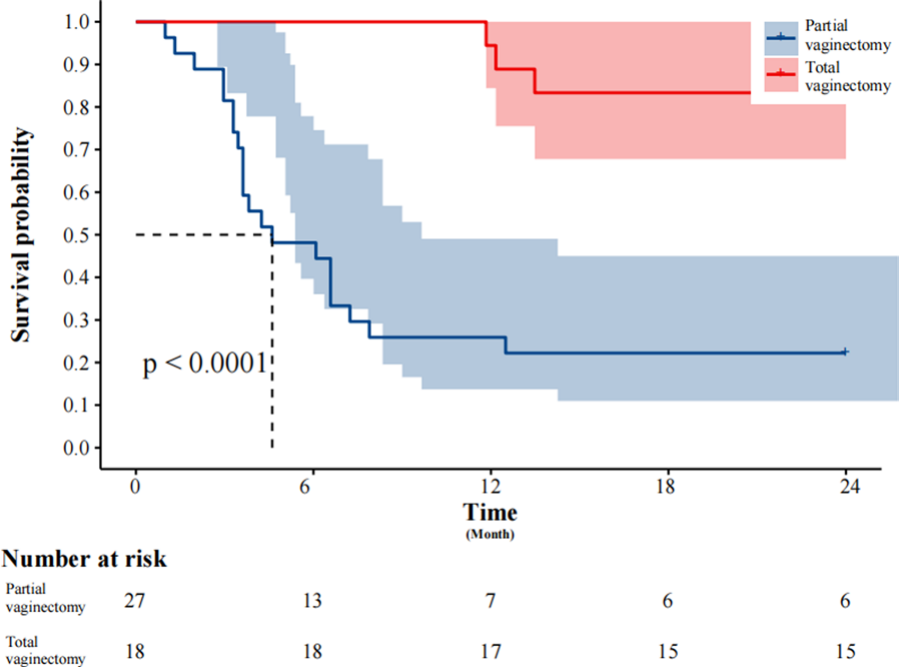
The Kaplan–Meier survival curve demonstrates the 2-year disease-free survival rates of patients with primary malignant melanoma of the vagina and cervix who underwent total vaginectomy

**Fig. 5:**
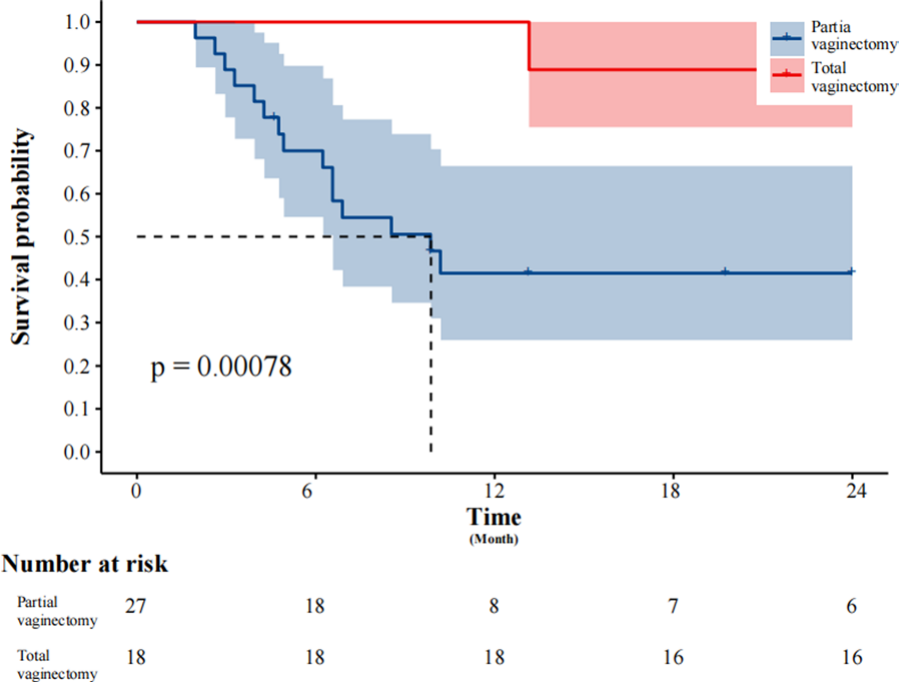
The Kaplan–Meier survival curve demonstrates the 2-year overall survival rates of patients with primary malignant melanoma of the vagina and cervix who underwent partial vaginectomy

The K-M survival analysis log-rank test stratified patients based on surgical approach and total vaginectomy. The 2-year DFS rate in the MIS group (68.75%) was significantly better than that in the open surgery group (34.48%) (*P* = 0.03) (Fig. 2). However, no significant difference in 2-year OS was observed between the groups (*P* = 0.078) (Fig. 3). Patients who underwent total vaginectomy showed significantly better survival outcomes compared to those with partial vaginectomy, as evidenced by the 2-year DFS (83.33% vs. 22.22%; *P* < 0.0001) (Fig. 4) and 2-year OS rates (88.89% vs. 22.22%; *P* = 0.001) (Fig. 5).

**Fig. 6:**
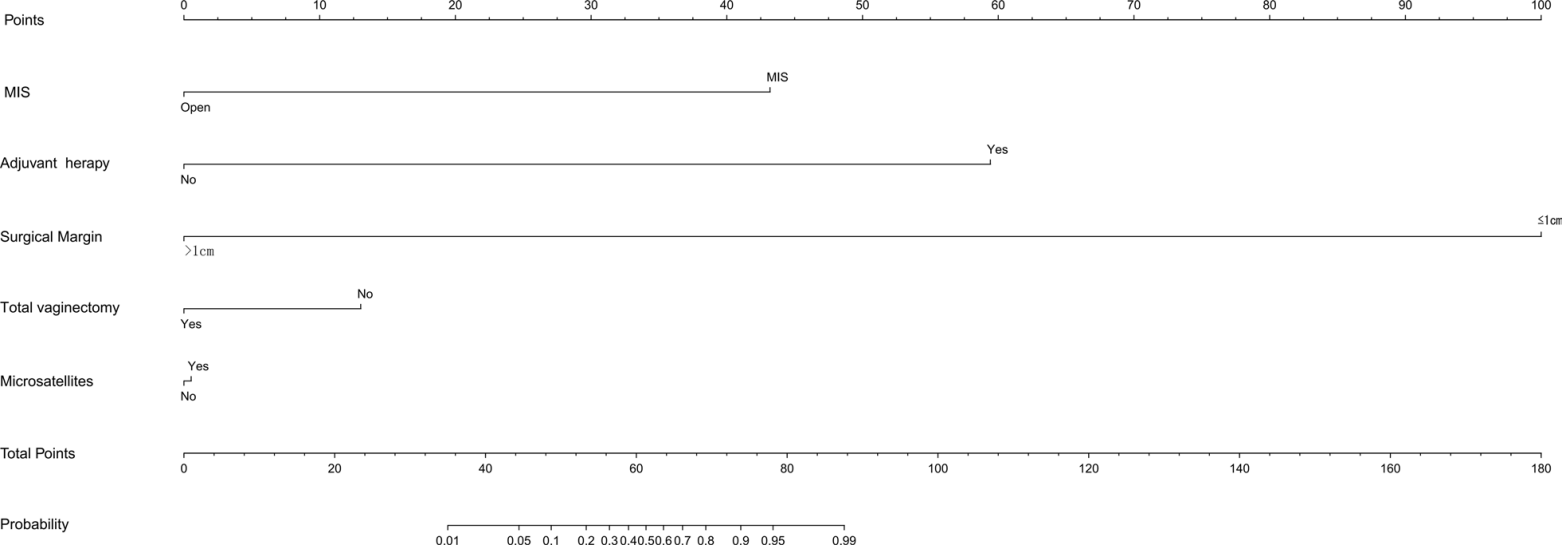
A nomogram to predict the risk of recurrence in primary malignant melanoma of the vagina and cervix

**Fig. 7:**
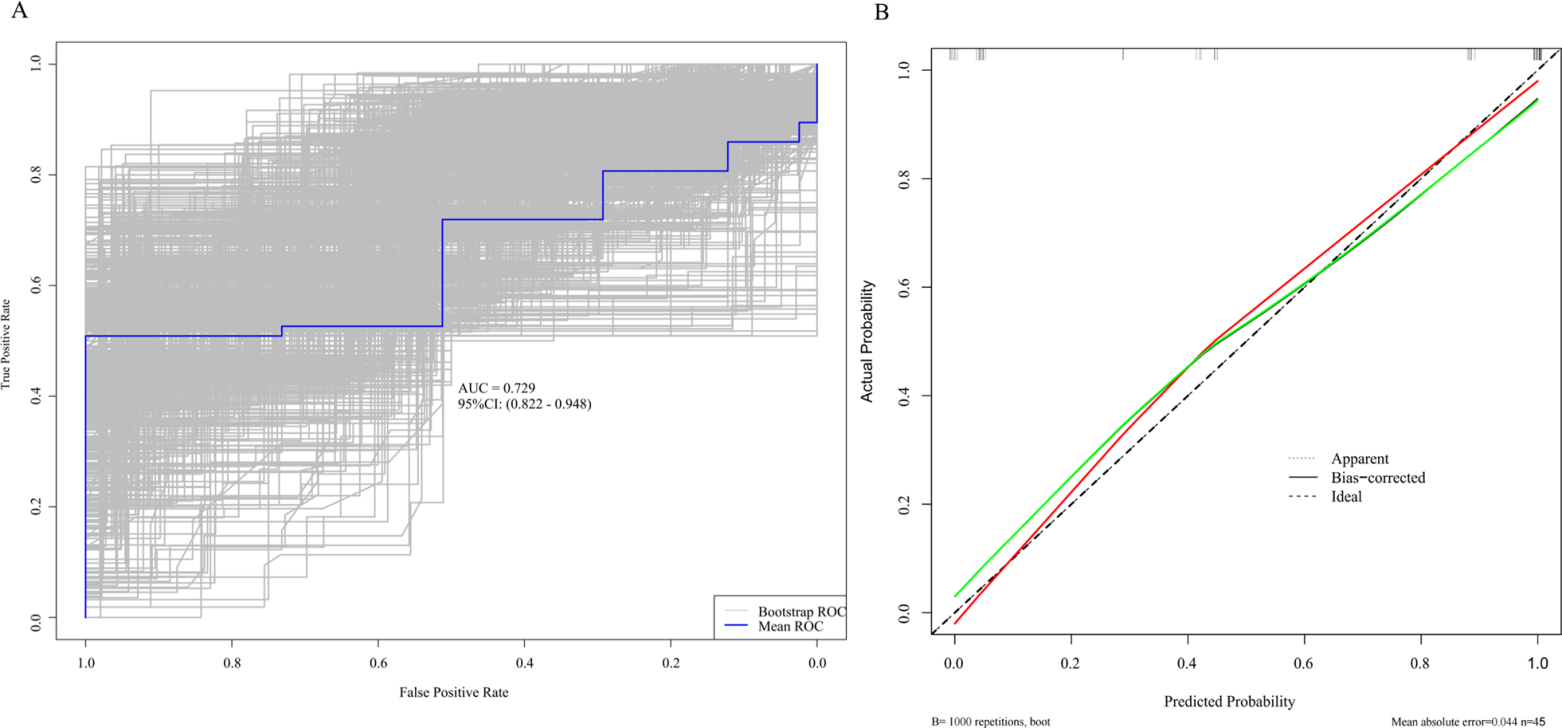
Receiver operating characteristics (ROC) curve and calibration plot. **A** Receiver operating characteristics (ROC) curve, **B** calibration plot

After conducting the aforementioned statistical analysis, in conjunction with findings derived from clinical practice, sensitivity analysis, and stratified analysis, we have successfully developed an innovative nomogram model that facilitates the comprehensive evaluation of clinical prognostic risk (Fig. 6). Due to the limited sample size and lack of an external validation dataset, we employed 1,000 bootstrap resampling iterations to validate both the multifactorial results and the nomogram. This involved constructing BS-ROC curves and calibration curves. Our nomogram demonstrated exceptional predictive performance, as evidenced by an AUC of 0.729 (95% CI from 0.822 to 0.948). The calibration curve exhibited a negligible average absolute error of 0.044, indicating close correspondence between our nomogram’s predictions and actual outcomes (Fig. 7).

## Discussion

The incidence of PMMC and PMMV, rare malignancies in the female reproductive tract, is exceptionally low, representing only 0.2–1% of all malignant melanomas [12]. In this study, our oncology center encountered a limited number of cases (45 cases) for initial surgical treatment over approximately 21 years, comprising 13 PMMCs and 32 PMMVs. These diseases are not only exceedingly rare but also have a poor prognosis, with a 5-year survival rate ranging from 5 to 25% [13]. Previous studies often combined vulvar malignant melanoma with PMMV [2, 14–16]; however, these conditions differ significantly in tumor biological characteristics, incidence, survival rates, and treatment approaches [2, 17]. The primary lesions of PMMC and PMMV often overlap in location and share similar tumor biological characteristics. Patients with PMMC and PMMV have a worse prognosis compared to those with vulvar malignant melanoma [17]. Therefore, we excluded vulvar malignant melanoma from our study and combined PMMC and PMMV cases for retrospective analysis. Currently, well-defined treatment guidelines are scarce. In addition to drawing from initial management strategies for cutaneous malignant melanoma, therapeutic approaches for vaginal and cervical cancer can also provide valuable insights. The primary treatment for newly diagnosed patients without distant metastasis remains surgical intervention [15].

Our aim is to provide a practical treatment approach for the initial management of PMMC and PMMV using real-world data. Recently, with advancements in MIS technology, its application in malignant tumor management has expanded. However, the 2019 LACC study results sparked debate over MIS for gynecological cancers like cervical and endometrial cancer, leading to restrictions in its use [18–20]. Some researchers suggest a possible link between the use of uterine manipulators in MIS and postoperative cervical/endometrial cancer recurrence [21, 22]. In our center, manipulator-free techniques are preferred for PMMV MIS, with uterine manipulators used in only two PMMC MIS cases. Melanoma’s higher malignancy degree leads to poorer DFS and OS compared to other gynecological cancers. Thus, factors influencing recurrence in other gynecologic malignancies during MIS may not affect PMMC and PMMV recurrence within 2 years, though further studies are needed for verification. In our study, total vaginectomy via MIS showed less perioperative blood loss and shorter hospital stays, while maintaining comparable postoperative complication rates, 30-day readmissions, and 2-year recurrence rates. These findings highlight MIS’s benefits in promoting rapid recovery.

Moreover, MIS showed superior 2-year DFS compared to open surgery. Our analysis indicates that this may be due to the higher incidence of total vaginectomy in the MIS group. Total vaginectomy, surgical negative margin length, and microsatellite metastases were identified as risk factors for recurrence in PMMC and PMMV patients. Additionally, in the subgroup analysis, significant improvements in 2-year DFS and OS were observed in patients undergoing total vaginectomy versus those with partial vaginectomy.

The retrospective design of this study is inevitably susceptible to selection bias and confounding factors. To mitigate the impact of these factors, we initially developed rigorous inclusion and exclusion criteria. Additionally, in order to address the potential issue of reverse causality, we constructed supplementary models (Models 1 and 2) for further analysis. Given the anatomical complexity and specific location of vaginal and cervical malignant melanoma lesions, achieving adequate negative surgical margins is challenging, particularly for lesions in the lower third of the vagina. This anatomy increases the risk of microsatellite and skip metastasis. Therefore, we excluded this factor from Model 1 to avoid confounding our analysis of recurrence risk. The findings from Model 2 align with those from the primary model; however, subtle discrepancies are observed in Model 1. Therefore, based on the results obtained from all three models, a novel nomogram was formulated to predict the recurrence risk of PMMC and PMMV. It should be noted that our study was limited by a small sample size due to the rarity of PMMC and PMMV cases. To ensure the robustness of our findings despite this limitation, we employed bootstrap statistical methodology for validation purposes. This approach effectively mitigated potential biases arising from small sample sizes and uneven sample distribution while fully demonstrating the reliability of our main model.

Historically, surgical approaches for PMMV and PMMC included radical resection, wide local excision, or partial vaginal resection [16]. However, compared to other treatments like radiotherapy and chemotherapy, neither conservative nor radical surgery has consistently yielded satisfactory oncologic outcomes. The effectiveness of radical surgery in improving PMMV prognosis has been debated, leading to controversy over the years [23]. This might be due to the lack of clarity regarding what constitutes radical surgery and confounding factors increasing recurrence risks in such interventions. Our study’s findings align with these observations. Our data showed that radical surgery influenced DFS in PMMV in univariate analysis, but this effect was not statistically significant in multivariate analysis, possibly due to these factors. Our study also revealed significant associations between microsatellite metastasis and negative margin distance with recurrence, highlighting the inadequacy of only excising visible tumors. The presence of skip metastases and undetectable residual lesions may contribute to tumor progression post-surgery [24]. Given malignant melanoma’s aggressiveness compared to other cancers, an appropriate surgical scope is essential to completely eliminate remaining tumors. In the management of cutaneous malignant melanoma, achieving a negative margin is crucial [25, 26]. Our study further confirms that a negative margin exceeding 1 cm significantly reduces the recurrence rate within two years. Research indicates that PMMV lesions often occur in the lower part of the vagina and may involve the labia minora, suggesting that total vaginectomy is effective in achieving adequate negative margins [2, 27]. Early diagnosis is essential for providing patients with the opportunity for radical surgical intervention. For example, hysteroscopy can accurately detect occult lesions of the cervix and endometrium, thereby enabling timely and appropriate treatment [28, 29]. Recent advancements in adjuvant therapy, including immunomodulatory drugs and monoclonal antibodies, as well as ongoing clinical trials, have shown promise in extending overall survival rates in tumor outcomes [30–32]. This highlights the importance of initial surgery in achieving radical treatment. Todo et al.’s study also suggested that patients with PMMV may benefit from initial surgical intervention performed with sufficient thoroughness [8]. Frumovitz’s study also found that early PMMV patients undergoing radical surgery had significantly longer survival than those receiving non-surgical treatment. Our study supports this finding. Despite no difference in postoperative adjuvant therapy usage between groups, MIS showed a significant advantage in postoperative treatment drug selection over open surgery. This is attributed to MIS’s increased use in the last five years and its combination with initial radical surgery, contributing to better 2-year DFS. Hence, radical resection for PMMV and PMMC should include complete vaginal resection and the excision of the urethra, vulva, and any tumor-involved bladder, rectum, or anus. This approach reduces the risk of local recurrence due to microsatellite and skip metastasis. Surgical procedure selection should be carefully assessed by a gynecologic oncologist based on preoperative staging, with total vaginal resection recommended along with resection of suspected involved organs.

However, our findings did not show a significant association between lymph node metastasis and recurrence, diverging from previous studies [13, 15]. This difference could be due to the inclusion of patients in early clinical stages. Additionally, excluding patients with distant metastases is reasonable since most with preoperative lymph-node metastases also had distant spread. A considerable number of surgeries at our center involve detailed lymph node dissection in areas like the pelvis and groin, potentially reducing the likelihood of nodal pathway recurrence.

Our study has inherent limitations. It is retrospective and conducted at a single center. Given the disease’s rarity, the sample size is small; however, as the leading malignant melanoma research center in southeast China, our hospital admits a substantial number of patients with this condition. The retrospective nature and lack of a standardized protocol for postoperative adjuvant therapy might introduce bias in survival data. The absence of certain data hinders the possibility of conducting statistical analysis. In the future, we plan to continue comprehensive patient follow-up, comparing 3- and 5-year DFS and OS rates among different surgical approaches. We also aim to conduct multi-center studies to increase sample size and reduce data bias.

## Conclusion

In conclusion, our study focused on the initial surgical treatment options for the rare gynecologic malignancies PMMC and PMMV, aiming to provide evidence-based guidelines for managing these uncommon tumors. With advancements in postoperative adjuvant therapy and ongoing clinical trials, radical surgery has emerged as an effective approach for improving tumor outcomes, surpassing the limitations of conservative surgery observed in previous years. Although no superior oncological outcome was achieved with the surgical approach in terms of DFS, total vaginectomy and wider surgical margins were found to be positively associated with a better prognosis. However, it should be noted that minimally invasive surgery may result in higher rates of total vaginectomy and longer negative margins. Therefore, we conclude that a combination of MIS and radical vaginal resection can serve as an effective initial surgical strategy for managing PMMV and PMMC. Nevertheless, further research involving larger sample sizes and longer follow-up periods is warranted to validate these findings.

## Supplementary Information

Supplementary material 1.

Supplementary material  2.

## Data Availability

The dataset(s) supporting the conclusions of this article is(are) included within the article (and its additional file(s)).
