# Supplementary material for: The clinical practice and outcomes of minimally invasive surgery in primary malignant melanoma of the vagina and cervix patients: a retrospective cohort study
Source: Orphanet J Rare Dis. 2025 Jun 6;20:286. doi: 10.1186/s13023-025-03760-x (PMC12144728; doi:10.1186/s13023-025-03760-x)
Supplement: Supplementary file 1 — Supplementary material 1. [file 13023_2025_3760_MOESM1_ESM.doc]

**Table S1** **Multivariate cox regression analysis of DFS.**

| **Characteris** | **DFS** | | | | | | |
| --- | --- | --- | --- | --- | --- | --- | --- |
|  | **Univariate** | **Analysis** |  |  | **Multivariate** | **Analysis** |  |
|  | **HR** | **95%CI** | ***P*-value** |  | **HR** | **95%CI** | ***P*-value** |
| **Age,year**  (<55 vs.≥55) | 0.322 | 0.478-2.801 | 0.748 | |  |  |  |
| **BMI,** **kg/m2**  (<22 vs. ≥22) | 0.312 | 0.486-2.707 | 0.974 | |  |  |  |
| **Comorbidity**  (Yes vs. No) | 1.049 | 0.663-3.886 | 0.294 | |  |  |  |
| **Type of hysterectomy**  (SH vs. RH) | 0.410 | 0.093-0.832 | 0.022﹡ | | 0.508 | 0.183-1.412 | 0.167 |
| **Lymphadenectomy**  (Yes vs. No) | 2.144 | 0.342-1.155 | 0.163 | |  |  |  |
| **LN metastasis**  (Yes vs. No) | 1.715 | 0.521-3.207 | 0.579 | |  |  |  |
| **AJCC stage**  (I and II vs.III) | 0.832 | 0.655-1.867 | 0.705 | |  |  |  |
| **Mitotic count**  (<10 vs.≥10) | 0.781 | 0.337-1.855 | 0.583 | |  |  |  |
| **Surgical approach**  (MIS vs. Open) | 2.850 | 0.686-5.126 | 0.221 | |  |  |  |
| Surgical Margin  (≤1cm vs.＞1cm) | 0.628 | 0.342-1.155 | 0.134 | |  |  |  |
| **Total vaginectomy**  (Yes vs. No) | 9.464 | 1.199-9.069 | 0.021﹡ | | 0.155 | 0.037-0.641 | 0.023﹡ |
| **Histologic subtype**  (Other vs.Spreading  And Nodular) | 1.028 | 0.548-1.742 | 0.936 | |  |  |  |
| **Number of tumors**  (Single vs. Multiple) | 0.766 | 0.315-1.781 | 0.512 | |  |  |  |
| **Microsatellites**  (Yes vs. No) | 3.120 | 0.006-0.139 | 0.000﹡ | | 2.893 | 1.042-8.029 | 0.000﹡ |
| **Adjuvant therapy**  (Yes vs. No) | 1.431 | 0.008-0.432 | 0.006﹡ | |  |  | 0.011﹡ |

﹡Statistically signifcant. *DFS* disease-free survival, *MIS* minimally invasive surgery, *BMI* body mass index, *RH* radical hysterectomy, *SH* simple hysterectomy.
