# Supplementary material for: The clinical practice and outcomes of minimally invasive surgery in primary malignant melanoma of the vagina and cervix patients: a retrospective cohort study
Source: Orphanet J Rare Dis. 2025 Jun 6;20:286. doi: 10.1186/s13023-025-03760-x (PMC12144728; doi:10.1186/s13023-025-03760-x)
Supplement: Supplementary file 2 — Supplementary material 2. [file 13023_2025_3760_MOESM2_ESM.doc]

**Table S2** **Multivariate cox regression analysis of DFS.**

| **Characteris** | **DFS** | | | | | | |
| --- | --- | --- | --- | --- | --- | --- | --- |
|  | **Univariate** | **Analysis** |  |  | **Multivariate** | **Analysis** |  |
|  | **HR** | **95%CI** | ***P*-value** |  | **HR** | **95%CI** | ***P*-value** |
| **Age,year**  (<55 vs.≥55) | 0.809 | 0.345-1.893 | 0.625 | |  |  |  |
| **BMI,** **kg/m2**  (<22 vs. ≥22) | 0.940 | 0.405-2.178 | 0.885 | |  |  |  |
| **Comorbidity**  (Yes vs. No) | 1.324 | 0.565-3.100 | 0.518 | |  |  |  |
| **Type of hysterectomy**  (SH vs. RH) | 0.343 | 0.126-0.932 | 0.036﹡ | | 0.438 | 0.135-1.420 | 0.169 |
| **Lymphadenectomy**  (Yes vs. No) | 2.256 | 0.918-5.546 | 0.076 | |  |  |  |
| **LN metastasis**  (Yes vs. No) | 1.742 | 0.729-4.162 | 0.212 | |  |  |  |
| **AJCC stage**  (I and II vs.III) | 1.279 | 0.760-2.155 | 0.354 | |  |  |  |
| **Mitotic count**  (<10 vs.≥10) | 0.784 | 0.338-1.817 | 0.571 | |  |  |  |
| **Surgical approach**  (MIS vs. Open) | 2.762 | 1.016-7.508 | 0.046﹡ | | 1.861 | 0.550-6.301 | 0.318 |
| Surgical Margin  (≤1cm vs.＞1cm) | 0.024 | 0.005-0.115 | 0.000﹡ | | 0.041 | 0.007-0.221 | 0.000﹡ |
| Lesion localization  (Upper vs. Middle  And Lower) | 0.703 | 0.300-1.648 | 0.418 | |  |  |  |
| **Total vaginectomy**  (Yes vs. No) | 0.042 | 0.006-0.314 | 0.002﹡ | | 0.081 | 0.007-0.961 | 0.046﹡ |
| **Histologic subtype**  (Other vs.Spreading  And Nodular) | 1.117 | 0.622-2.003 | 0.711 | |  |  |  |
| **Number of tumors**  (Single vs. Multiple) | 0.509 | 0.280-0.927 | 0.027﹡ | | 0.754 | 0.285-1.998 | 0.571 |
| **Microsatellites**  (Yes vs. No) | 3.548 | 1.378-9.141 | 0.009﹡ | | 4.066 | 1.162-14.231 | 0.028﹡ |
| **Adjuvant therapy**  (Yes vs. No) | 1.325 | 0.554-3.171 | 0.527 | |  |  |  |

﹡Statistically signifcant. *DFS* disease-free survival, *MIS* minimally invasive surgery, *BMI* body mass index, *RH* radical hysterectomy, *SH* simple hysterectomy.
